# Patterns of Mental Health Service Use in Australian Workers with Low Back Pain: A Retrospective Cohort Study

**DOI:** 10.1007/s10926-024-10180-4

**Published:** 2024-02-24

**Authors:** S. E. Gray, M. Di Donato, L. R. Sheehan, R. Iles, A. Collie

**Affiliations:** Healthy Working Lives Research Group, School of Public Health and Preventive Medicine, Monash University, Melbourne, Australia

**Keywords:** Low back pain, Workers compensation, Mental health, Health services research

## Abstract

**Purpose:**

To describe the volume, timing and provider of mental health services provided to workers with accepted low back pain (LBP) claims, and to identify determinants of service volume and time to first mental health service.

**Methods:**

Using claim and service-level workers’ compensation data from four Australian states (Queensland, South Australia, Western Australia, Victoria) for LBP claims with at least one mental health service lodged between 1 July 2011 and 30 June 2015. Mental health services occurring 30 days prior to 730 days following claim acceptance were examined. Outcomes were number of mental health services and time (weeks) from claim acceptance to first service, calculated overall, by provider and interaction type, and by independent variables (age group, sex, time loss duration, financial year of lodgement, jurisdiction, socioeconomic status, remoteness). Negative binomial and Cox regression models examined differences between service volume and time to first service by independent variables, respectively.

**Results:**

Of workers with LBP claims who accessed mental health services, psychologist services were most common (used by 91.2% of workers) and 16% of workers saw multiple provider types. Number of services increased with time loss duration, as did time to first service. Victorian workers had the most services, yet accessed them latest.

**Conclusions:**

Psychologist services were most common, longer duration claims used more mental health services but accessed them later, and there were a number of jurisdictional differences. Results suggest opportunities for workers’ compensation authorities to provide, to those who may benefit, greater and earlier access to mental health care.

**Supplementary Information:**

The online version contains supplementary material available at 10.1007/s10926-024-10180-4.

## Introduction

Low back pain (LBP) is a leading public health issue that can limit both quality of life and work performance [1]. In 2020 LBP contributed to an estimated 69 million years lived with disability globally [2], largely among working age people [3]. While most people recover from an episode of low back pain relatively quickly, the condition is often recurrent and some episodes can become chronic (i.e., persist beyond three months) [4, 5]. Chronic LBP and poor mental health are strongly linked [6–8]. Mental health conditions such as depression and anxiety can either contribute to LBP or develop as a consequence of LBP [9]. Enduring pain, side effects of medication, loss of independence, social isolation, and engagement with stressful administrative processes (in the event of compensable LBP) [9–11], can all be drivers of poor psychological health in people with LBP, who may report symptoms such as general sadness, anger, sleep problems, and reduced drive and engagement [1, 4].

Research has shown an increased risk for mental health problems in people with musculoskeletal conditions and injuries [7, 12], with prior studies finding that those with an injury had more than three times the risk of mental health-related hospitalisations and 1.5 times the risk of a mental health-related physician visit compared to a non-injured cohort, after adjusting for comorbidities and pre-existing mental health service use [13]. Studies have shown up to 25% of those with an occupational musculoskeletal injury report moderate to severe psychological distress [10, 12, 14, 15], with the prevalence higher in those with acute LBP [16]. Despite the prevalence of psychological distress, these same studies report underutilisation of mental health services. A survey of Australian workers’ compensation claimants observed only one-fifth with moderate distress and two-fifths with severe distress reported accessing mental health services in the previous month [10]. Another Australian study in the state of Victoria found that whilst a third of injured workers experienced severe mental health problems, only 41.4% accessed mental health services within the 18-month follow-up [17]. Little is known, however, about the nature or patterns (e.g., duration, timing, intensity) of the services actually provided.

People with longer duration claims, which commonly involve mental health [18], experience the worst outcomes and are the more costly. Knowing more about the patterns of mental health service use would help identify gaps in service delivery such as treatment delays, understand the volume and types of services currently being delivered and by whom, and identify service variation. This knowledge could support mental health care service planning and policy in workers’ compensation schemes, especially as literature supports early intervention to achieve better outcomes [5], and that mental health services can improve recovery [19]. This study aims to describe the volume, timing and type of mental health services provided to workers with accepted low back pain claims. A second aim is to identify (demographic and claim-related) determinants of mental health service volume and time to first compensated mental health service.

## Methods

### Setting

In Australia, there are eleven major workers’ compensation schemes: one for each state or territory and three for national employers and industries. Wage replacement and ‘reasonable and necessary’ healthcare and service expenses are provided for workers where injury or illness can be attributed to employment. Workers’ compensation may also fund mental health services for workers with an accepted claim for a mental health condition, or a worker with another injury or condition where the workers’ compensation scheme determines that such services will support return to work and rehabilitation. Approval for mental health services may be required from a claims manager if the primary compensable condition is physical. Mental health services in Australia can also be funded publicly (i.e., via Medicare, Australia’s national health insurance scheme), via private health insurance or by an individual directly (i.e., “out-of-pocket”). Eligibility for public funding may be granted by a General Practitioner (i.e., a Primary Care Physician) via a mental health care plan and referral (up to 5 treatments per calendar year at the time of the study) [20]. Workers’ compensation scheme funding for mental health services therefore offers an alternative funding source for those whose injury is work-related. Choice of mental health professional is at the discretion of the injured worker, provided they are registered with both the relevant national healthcare registering body (e.g., Australian Health Practitioner Regulation Agency) and the workers’ compensation regulator.

### Data Source

Data from the Monash University Multi-Jurisdictional Workers’ Compensation Database (MJD) were used. This database contains de-identified administrative workers’ compensation claim and service payments information for musculoskeletal conditions from five Australian workers’ compensation jurisdictions [21]. The MJD contains accepted claims made by workers that were lodged between 1 July 2010 and 30 June 2015 from Victoria, Queensland, Western Australia, South Australia and Comcare (the national scheme covering federal government employees and some large national employers) for LBP, limb fracture and non-specific limb conditions. Details on funded health services are also included (e.g., date of service, provider type, interaction type, provider ID), linked to claims data by a unique identifier. The database and its development have been described elsewhere [21].

### Inclusion Criteria

This study included LBP claims from Victoria, Queensland, South Australia and Western Australia if lodged by the employer between 1 July 2011 and 30 June 2015. These jurisdictions were selected as their health services data contained sufficient information to identify mental health services. The Victorian and South Australian workers’ compensation schemes, at the time of this study, utilised a two-week excess period in which the employer must fund time loss. To account for this, only claims with two weeks or longer wage replacement from Queensland and Western Australia were included. The Type of Occurrence Classification System [22] was used to identify LBP claims and details are provided in Supplementary Table 1.

Mental health services were defined as “an interaction between a mental health professional and a compensated worker”. To identify eligible services, a list of services from each jurisdiction were assessed by two reviewers independently according to the above definition. Mental health services data were then categorised by provider type (‘Psychiatrist’, ‘Psychologist’, ‘Social worker, counsellor or rehabilitation counselling’ [mental health-specific] or ‘Other and unspecified’ [where a service was clearly mental health related, but the provider type could not be identified]) and interaction type (‘Single/one-on-one consultation’, ‘Group consultation or therapy’, or ‘Other interaction type’). A third reviewer acted as adjudicator where discrepancies in allocating services to specific categories occurred.

Only workers with claims for a mental health service were included as the focus is on the patterns of mental health service use for those that received them. Prevalence of mental health service use has been explored previously [18]. Mental health services that occurred up to 30 days prior (as services can be funded retrospectively) to 730 days following claim acceptance (as workers have access to services in all jurisdictions within this time frame) were included.

Duplicate records were excluded to ensure only one type of interaction with a particular provider per person per day occurred. For example, two one-on-one psychologist consultations were not considered feasible yet a one-on-one consultation AND a group consultation with a psychologist on one day was considered feasible. Less than 1% of services were for Other and unspecified providers or Other interaction types, and were therefore not included in analyses.

### Outcome

The outcomes of interest were (i) the number of mental health services per claim and (ii) the time (in weeks) from claim acceptance to first service. These were calculated overall, by mental health service provider, by interaction type, and by independent variables.

### Independent Variables

Covariates statistically associated with workers’ compensation claim outcomes in prior studies and that were available in the dataset were included [23]. Date of lodgement was used to derive Australian financial year of lodgement (e.g., a claim lodged between 1 July 2012 to 30 June 2013 was coded to financial year 2013). Worker sex was already defined in the claims dataset as binary (male or female). Age at time of lodgement was categorised into 15–25 years, 26–35 years, 36–45 years, 46–55 years, 56–65 years and > 65 years age groups. Jurisdiction is the state workers’ compensation scheme in which the claim was lodged. Worker postcode was mapped to the Socio-Economic Index for Areas and Australian Statistical Geography Standard to derive the Index of Relative Socioeconomic Advantage and Disadvantage (IRSAD) and Accessibility/Remoteness Index of Australia (ARIA), respectively [24, 25]. Socioeconomic status (IRSAD) was grouped into most disadvantaged (lowest quintile), middle three quintiles and most advantaged (highest quintile). Remoteness was grouped into major cities of Australia, inner regional Australia, and outer regional/remote/very remote Australia.

We expected a strong covariance between duration of time loss and service volume (longer claim duration allows opportunity for more services). Further, with increased claim length exposure to stressors (e.g., social or financial effects of claiming or injury, workers’ compensation processes, independent medical evaluations) may also increase [26, 27]. Therefore, duration of time loss was used as a covariate to enable comparison of outcomes by time loss durations and categorised into groups that represent typical claim milestones (such as a reduction in the amount of pre-injury earnings paid): 2 to 13 weeks; 13 to 26 weeks; 26 to 52 weeks; 52 to 76 weeks, and; 76 + weeks.

### Analysis

Claim information was combined with services (one record-to-many). The total number of claims and mental health services, along with the proportion of all claims and mental health services were tabulated. Median number of services and time to first mental health service (in weeks) were also calculated overall and by provider type and by independent variables. All medians were reported with corresponding interquartile ranges. Figures were developed showing both the density of services and the number of services over the two-year follow-up period by provider type. Supplementary Fig. 1 also shows this density by duration of time loss group, separated by provider type.

The services dataset was then aggregated to one record per claim, retaining all claim and outcome information. A Venn diagram was developed showing the number of claims that received services from each combination of provider type (e.g., psychologist and psychiatrist). Negative binomial regression models were developed to examine statistical differences between total number of mental health services by independent variables, as Poisson regression was considered inappropriate due to overdispersion. The coefficient was exponentiated and expressed as an incidence rate ratio (IRR) with corresponding 95% confidence intervals (statistically significant if confidence interval does not include one). Cox regression was performed to examine statistical relationships between time to first mental health service and independent variables. Results were expressed as hazard ratios (HRs) with corresponding 95% confidence intervals.

Missing age, remoteness and socioeconomic information was imputed using multiple imputation (multivariate imputations by chained equations, five iterations) for both regression models. Statistical significance was set at *p* < 0.05. All analyses were conducted using R Version 4.0.3 (Vienna, Austria). Monash University Human Research Ethics Committee approved the project (ID: 17,267, November 2018).

## Results

Less than 10% of LBP claims met the inclusion criteria of having had a mental health service funded by workers’ compensation [18]. Combined, these claims recorded a total of 30,495 mental health services (Table 1). More than half of the cohort had at least 76 weeks of compensated time loss, and almost two-thirds were male (64.9%). Two-thirds of mental health services were utilised by those with at least 76 weeks’ time loss over the course of their claim. More than half of all services were provided to workers in Victoria (54.3%) followed by Queensland (30.2%).

### Total Services

Table 1 shows that there was a higher median number of services for psychiatrists than psychologists or social workers (in those who saw that provider type at least once). With increasing time loss, the median number of services increased. Workers injured in Victoria had a significantly higher number of services than other jurisdictions. After adjustment for other factors in the regression model, workers injured in the two most recent years had significantly more services (IRR 1.28 [95% CI 1.16, 1.41] and IRR 1.23 [95%CI 1.11, 1.36] for 2014 and 2015, respectively), and females had a significantly higher number of services than males (IRR 1.12 [95% CI 1.04, 1.21]). Those living in inner regional Australia had significantly fewer services than those in major cities.

**Table 1:** Description of cohort with volume and proportion of claims and services, median number of services by independent variable and negative binomial regression results showing the rate of any mental health service use

	Total claims	% of all claims	Total services	% of all services	Median number of services (IQR)	IRR (95% CI)	*p*-value
Overall	2800	100	30,495	100	20 (10, 35)		
Provider type^† ^^							
Psychiatrist	624	22.3	5334	17.5	28 (16, 50)		
Psychologist	2554	91.2	24,885	81.6	19 (10, 32)		
One-on-one consultation	2,547	91.0	22,700	74.4	18 (9, 33)		
Group consultation	248	8.9	2185	7.2	23 (17, 31)		
Social work/Other counselling	91	3.3	276	0.9	9 (4, 21)		
Time loss duration							
< 13 weeks	126	4.5	657	2.2	7 (5, 16)	0.37 (0.31, 0.44)	< 0.001
13 to 26 weeks	251	9.0	1329	4.4	8 (4, 13)	0.38 (0.33, 0.44)	< 0.001
26 to 52 weeks	544	19.4	4084	13.4	11 (6, 25)	0.54 (0.49, 0.59)	< 0.001
52 to 76 weeks	395	14.1	4199	13.8	19 (10, 29)	0.80 (0.72, 0.89)	< 0.001
76 + weeks	1484	53	20,226	66.3	24 (13, 40)	Ref	
Financial year							
2012	693	24.8	6783	22.2	20 (9, 35)	Ref	
2013	700	25.0	7423	24.3	19 (10, 39)	1.10 (0.99, 1.21)	0.068
2014	728	26.0	8394	27.5	21 (10, 36)	1.28 (1.16, 1.41)	< 0.001
2015	679	24.3	7895	25.9	20 (11, 32)	1.23 (1.11, 1.36)	< 0.001
Sex							
Female	982	35.1	10,953	35.9	21 (10, 35)	1.12 (1.04, 1.21)	0.002
Male	1818	64.9	19,542	64.1	20 (10, 35)	Ref	
Age group							
15–25 years	256	9.1	2519	8.3	19 (10, 32)	0.87 (0.76, 0.99)	0.038
26–35 years	720	25.7	7520	24.7	19 (10, 35)	0.97 (0.89, 1.07)	0.574
36–45 years	842	30.1	9614	31.5	22 (11, 35)	Ref	
46–55 years	727	26.0	7900	25.9	20 (10, 36)	0.91 (0.83, 1.00)	0.048
56 + years	255	9.1	2942	9.6	21 (11, 34)	0.92 (0.81, 1.05)	0.211
Jurisdiction							
Queensland	924	33.0	9207	30.2	19 (8, 38)	0.85 (0.78, 0.93)	< 0.001
South Australia	359	12.8	2194	7.2	11 (6, 19)	0.40 (0.36, 0.45)	< 0.001
Victoria	1117	39.9	16,544	54.3	24 (14, 38)	Ref	
Western Australia	400	14.3	2,550	8.4	10 (6, 18)	0.44 (0.39, 0.49)	< 0.001
Socioeconomic status*							
Most advantaged quintile	399	14.3	4299	14.1	19 (10, 36)	0.98 (0.89, 1.09)	0.747
Second to fourth quintiles	1804	64.4	20,165	66.1	21 (10, 36)	Ref	
Most disadvantaged quintile	472	16.9	5112	16.8	20 (11, 34)	0.97 (0.89, 1.07)	0.589
Remoteness**							
Major cities of Australia	2027	72.4	23,172	76.0	21 (11, 36)	Ref	
Inner regional Australia	449	16.0	4570	15.0	20 (9, 34)	0.88 (0.80, 0.96)	0.006
Outer regional/remote/very remote Australia	198	7.1	1828	6.0	18 (8, 35)	0.99 (0.87, 1.14)	0.924

*IRR* (incidence rate ratio) > 1 indicates higher number of mental health services than reference

*IQR *interquartile range

^†^Workers could consult with multiple providers, therefore will not add to 100% for provider type

*919 services with missing socioeconomic information on claim

^Medians only among those who utilised each provider type and service

**925 services with missing remoteness information on claim

### Provider Type

**Fig. 1:**
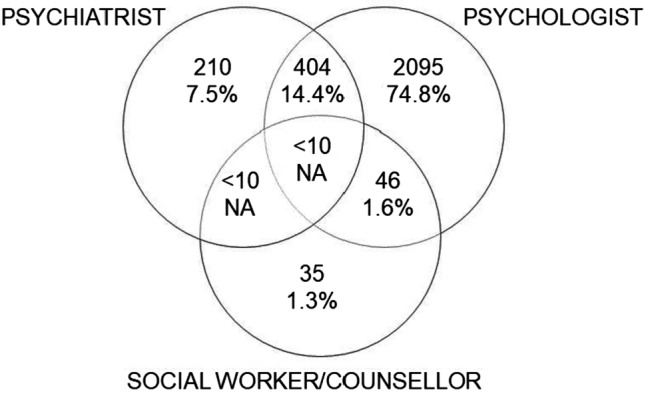
Number and percentage of all injured workers who visited each provider at least once

The majority of services (74.4%) were for a one-on-one consultation with a psychologist, with consultations to psychiatrists also common (17.5%) [Table 1]. Most workers only received services from psychologists (*n* = 2,095, 74.8%) (Fig. 1). Sixteen percent (*n* = 460) received services from multiple providers with 404 receiving services from both a psychiatrist and psychologist (14.4%), and less than ten people received services from all three provider types.

### Timing of Services

**Fig. 2:**
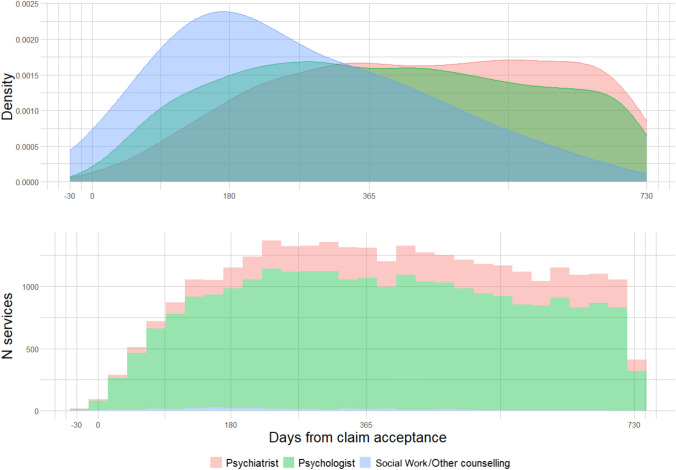
Distribution and frequency of service use by provider type. Density plot describes the distribution of each service independently and histogram shows the total volume of services every thirty days relative to the claim acceptance date

Figure 2 shows that peak usage of social work and other counselling services is earliest and peaks around 180 days then reduces steadily. Psychology increases more slowly, reaching a plateau at around 270 days then reduces more gradually from 455 days. Psychiatry services peak the latest at around 550 days (with high use between 365 days and 640 days). Psychological services were the most common service throughout the follow up period.

### Time to First Service

The median time to first service was shortest for psychiatrists. Median time to first service increased with increasing time loss duration (see Table 2). Queensland had the shortest median time to first service whereas Victoria had the longest. Those furthest from major cities had the shortest median time to first service. There were no obvious patterns for time to first service by sex, age group, financial year of lodgement or socioeconomic status. Cox regression results show that compared to those with time loss duration of at least 76 weeks, time to first service was generally shorter in line with shorter time loss duration, with those with 13–26 weeks’ time loss recording the shortest duration to first service. Workers in Queensland and Western Australia utilised services significantly earlier in their claim than those in Victoria.

**Table 2:** Time to first service by independent variable and Cox regression results

	Median time to first service in weeks (IQR)	HR (95% CI)	*p*-value
Overall	26.6 (13.3, 45.3)		
Provider type*			
Psychiatrist	21.4 (11.3, 37.2)		
Psychologist	27.7 (14.0, 47.7)		
One-on-one consultation	26.6 (13.3, 45.0)		
Group consultation	47.1 (27.0, 69.0)		
Social Work/Other counselling	22.1 (8.9, 36.1)		
Time loss duration			
< 13 weeks	12.3 (5.6, 36.3)	2.17 (1.79, 2.62)	< 0.001
13 to 26 weeks	13.7 (6.6, 28.1)	2.29 (1.98, 2.65)	< 0.001
27 to 52 weeks	20.2 (11.3, 34.1)	1.64 (1.47, 1.83)	< 0.001
53 to 76 weeks	21.9 (9.1, 35.0)	1.46 (1.30, 1.65)	< 0.001
76 + weeks	30.9 (17.0, 50.9)	Ref	
Financial year			
2012	26.9 (13.7, 43.4)	Ref	
2013	27.4 (14.0, 48.6)	0.98 (0.88, 1.09)	0.666
2014	25.9 (11.9, 43.3)	0.96 (0.86, 1.07)	0.436
2015	25.9 (13.3, 46.7)	1.02 (0.91, 1.14)	0.718
Sex			
Female	25.6 (13.1, 43.6)	1.01 (0.93, 1.09)	0.896
Male	26.9 (14.0, 46.3)	Ref	
Age group			
15–25 years	24.9 (11.1, 45.1)	1.08 (0.93, 1.25)	0.318
26–35 years	27.6 (14.3, 47.0)	0.95 (0.86, 1.05)	0.333
36–45 years	25.0 (13.0, 43.1)	Ref	
46–55 years	26.3 (13.1, 46.3)	0.90 (0.81, 0.99)	0.038
56 + years	29.0 (18.9, 47.2)	0.88 (0.76, 1.01)	0.077
Jurisdiction			
Queensland	17.4 (8.0, 29.1)	2.01 (1.82, 2.22)	< 0.001
South Australia	29.1 (14.3, 51.7)	1.07 (0.94, 1.23)	0.283
Victoria	32.7 (18.5, 53.3)	Ref	
Western Australia	27.9 (14.0, 45.1)	1.41 (1.25, 1.60)	< 0.001
Socioeconomic status*			
Most advantaged quintile	26.6 (14.0, 50.9)	0.95 (0.85, 1.07)	0.415
Second to fourth quintiles	26.9 (13.3, 45.1)	Ref	
Most disadvantaged quintile	25.1 (13.1, 44.0)	1.02 (0.92, 1.13)	0.713
Remoteness**			
Major cities of Australia	27.1 (14.4, 47.1)	Ref	
Inner regional Australia	24.3 (11.4, 41.3)	1.01 (0.91, 1.12)	0.879
Outer regional/remote/very remote Australia	21.4 (10.0, 41.1)	0.96 (0.82, 1.11)	0.552

*IQR *interquartile range

*HR *(hazard ratio) > 1 indicates faster time to first mental health service than reference

*Time to first service only among those who utilised each provider type and service

## Discussion

We observed that use of workers’ compensation-funded mental health services in workers with LBP claims is uncommon (9.7%) [18], there were long periods of time between claim acceptance and first mental health services, and most mental health services are used by workers with long periods of time off work. Among those who accessed funded mental health services, more than 90% saw a psychologist at least once, and 16% received care from multiple providers, with the most common combination involving psychologists and psychiatrists. Prior research using large self-reported surveys suggest mental health problems including psychological distress, depression and anxiety affect a greater proportion of workers with compensated musculoskeletal conditions than the 10% in our study [10, 15, 17]. Accordingly, there appears to be a gap between funded service provision (in the four jurisdictions described here) and what is reported by workers.

Clinical guidelines recommend health professionals treating LBP consider psychosocial components [28–30], as psychological distress can become the most significant issue for treatment and management [11, 31]. This is especially true for those with chronic LBP. Workers with chronic LBP may struggle with pain and coping, which is not conducive to return to work, and the longer a worker is absent from work the more social, mental and personal difficulties there are that present themselves [27]. The slower uptake of mental health services for those with long duration claims could possibly be due to a failure to identify psychological issues early on in people with LBP, or that psychological issues only become apparent as the LBP progresses [30, 32]. In order to prevent the development of persistent LBP and subsequent poor outcomes, identifying the need for funded mental health care as early as possible in the claim will possibly improve functional recovery and return to work outcomes.

It has been suggested that effective rehabilitation should focus on adaptation of both the worker and the environment (e.g., workplace) to their health and personal circumstances rather than the LBP itself [33]. There is strong evidence that implementing work-focused cognitive behavioural therapy (CBT) for those with mental health conditions can help reduce duration of work absence and costs associated with work disability [34, 35]. Work-focused CBT adapts traditional CBT by more explicitly focusing on work (or return to work), encouraging self-efficacy, and providing the injured worker with strategies to make positive cognitive and behavioural changes in their workplace [35]. This may include changing a stressful work situation by transferring to another role or department, or gradually returning to work.

The onset of mental health problems could also be a consequence of personal, healthcare-related, work-related or claim-related factors occurring during the course of the claim, that also affect recovery [36]. There may be an opportunity for improved screening to identify people at risk of developing mental health problems, in whom preventive strategies in addition to funded mental health care would be helpful. Such prevention strategies should target individual risk factors as well as attempt to reduce exposure to other processes that may be psychologically harmful, such as stressful claim processes, adverse employer responses to their injury, and lack of social support [26, 37].

More mental health services were used by those living in major cities, reflecting availability of specialist mental health services [20]. Workers’ compensation insurers could recognise this, and due to the increasing use of telehealth, suggest this is an option to ensure equitable access to mental health services. Females had a significantly higher number of services compared to males, consistent with the observed increased prevalence of distress following injury [12] and higher likelihood of help-seeking for mental health [38]. More mental health services were used in more recent years. This could reflect increased awareness and availability of psychological supports for injured workers, or that barriers to accessing compensated services were reduced, as insurers may be getting better at recognising who may benefit from mental health services. Further, it could represent improved mental health literacy or a reduction in the help-seeking stigma that often surrounds mental health. While access to mental health services is a major determinant of utilisation, a broader set of factors including mental health literacy, stigma, and healthcare, workplace, personal or insurance resources available to the individual are important to consider [39], yet these could not be accounted for using administrative data.

There were differences in outcomes between states, likely due to variation in legislation and operational structures between workers’ compensation jurisdictions [23]. Funded services for mental health professionals was earliest in Queensland whereas workers in the Victorian scheme accessed mental health services later in their claim. This suggests there is an opportunity for Victorian workers with LBP to be provided earlier access to mental health services, and that differences persist despite efforts to standardise between jurisdictions. Claims accepted in the Victorian workers’ compensation scheme may be more complex or severe due to the employer excess period where the employer is liable for the first ten days of incapacity and medical expenses to a nominal amount, and thus require more services, however South Australia also had a two-week employer excess during the study period with differing mental health service outcomes (such as fewer services). Mental health service use such as volume may also differ between jurisdictions, particularly for longer claims, as there are time variations for entitlement to benefits.

Claims processes and engagement with compensation schemes are known to be stressful, and can exacerbate psychological distress and slow recovery [26, 37]. Since the study period, Queensland and South Australia have introduced changes to provision of mental health services. A new initiative in Queensland screens injured workers early in the claim to determine whether particular supports, such as mental health services, may help improve recovery [40]. A voluntary mental health support service to help injured workers respond “as best as possible” to their change in circumstances and workers’ compensation claim has been set up in South Australia [41]. Thus, this study could be repeated with data since introduction of these initiatives to compare uptake of mental health services, their timing, and subsequent return to work outcomes.

This comprehensive study examines the patterns of compensated mental health service use among a cohort of workers with LBP, including total services and time to first service. This was achieved using an Australian-first large-scale administrative dataset of multiple workers’ compensation jurisdictions’ claims and services data. A limitation of the study is that we were only able to capture mental health services funded by workers’ compensation schemes, and of those only specialist mental health services were captured (e.g., no mental health plans developed with a General Practitioner/Primary Care Physician or mental health support offered by allied health professionals). Therefore, it is likely that workers engaged with more mental health services than were included in this study. A future study that links workers’ compensation data to Medicare data may help address this, however, privately funded services would still not be captured. There is a possibility that data were incorrectly coded, meaning some relevant services were omitted or incorrectly coded as mental health services. However, given these data are collected for the purposes of managing the scheme, it is expected to be representative. Furthermore, data used in this study were from 2011 to 2015, and workers’ compensation and health system policies and practices are dynamic, with legislative changes and new initiatives (as detailed earlier) occurring over time. Repeating this study with more recent data would allow comparison of outcomes before and after their introduction.

Addressing mental health has been found to promote recovery for those with LBP [42]. A valuable next step would be to determine the relationship between patterns of mental health service use and recovery, such as whether those with earlier psychological intervention had more positive health and return to work outcomes than those without it or who received it later in their claim. Findings suggest there are opportunities for psychological intervention for those experiencing occupational injury earlier in a claim, particularly in different jurisdictions.

## Conclusion

This study utilised service use data from four Australian workers’ compensation jurisdictions to show that psychologists were the most common mental health service provider for compensated workers and that mental health service usage occurs later in a claim. Workers with longer durations of time off work received more funded mental health care than those with shorter durations, and waited longer to receive their first funded mental health service. There were a number of jurisdictional differences, likely reflecting different policies and practices. Results suggest opportunities for workers’ compensation regulators and insurers to provide greater and earlier access to mental health care alongside physical treatment for those who would benefit.

## Supplementary Information

Below is the link to the electronic supplementary material.

Supplementary material 1 (DOCX 130.8 kb)

Supplementary material 2 (DOCX 12.5 kb)
