# Supplementary material for: Patterns of Mental Health Service Use in Australian Workers with Low Back Pain: A Retrospective Cohort Study
Source: J Occup Rehabil. 2024 Feb 24;34(4):913–22. doi: 10.1007/s10926-024-10180-4 (PMC11550282; doi:10.1007/s10926-024-10180-4)
Supplement: Supplementary file 1 — Supplementary material 1 (DOCX 130.8 kb) [file 10926_2024_10180_MOESM1_ESM.docx]

***Supplementary Figure 1* - Distribution of services over time by provider type and duration of time loss group**


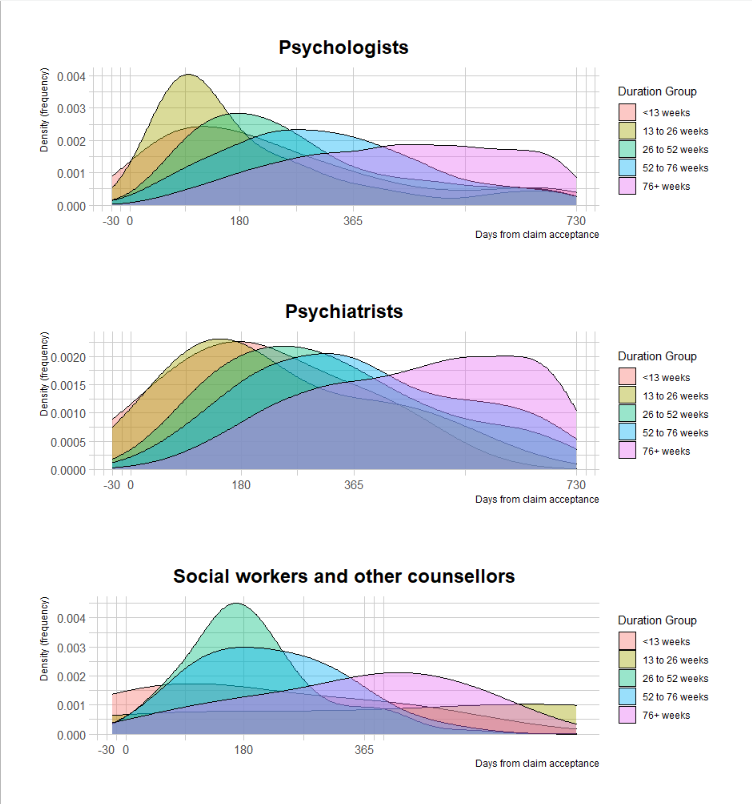


Supplementary Figure 1 - Distribution of services over time by provider type and duration of time loss group
