# Supplementary material for: Patterns of Mental Health Service Use in Australian Workers with Low Back Pain: A Retrospective Cohort Study
Source: J Occup Rehabil. 2024 Feb 24;34(4):913–22. doi: 10.1007/s10926-024-10180-4 (PMC11550282; doi:10.1007/s10926-024-10180-4)
Supplement: Supplementary file 2 — Supplementary material 2 (DOCX 12.5 kb) [file 10926_2024_10180_MOESM2_ESM.docx]

***Supplementary Table 1* – Low back pain selection criteria using Type of Occurrence Classification System 3^rd^ Edition**

| **Nature of injury** | AND | **Location of injury** |
| --- | --- | --- |
| 228 – Trauma to muscles and tendons, not elsewhere classified OR 229 – Trauma to muscles and tendons, unspecified OR  239 – Soft tissue injuries due to trauma or unknown mechanisms with insufficient information to code OR  422 – Disc displacement, prolapse, degeneration or hernia OR  459 – Back pain, lumbago, or sciatica OR  533 – Muscle, tendon strain (non-traumatic) |  | 311 – Lower back |
